# Heart failure patients demonstrate excellent 1-year outcomes after total knee arthroplasty despite high healthcare utilization

**DOI:** 10.1007/s00590-025-04549-1

**Published:** 2025-10-09

**Authors:** Nickelas Huffman, Abizairie Sánchez-Feliciano, Khaled A Elmenawi, Shujaa T Khan, Ignacio Pasqualini, Benjamin E Jevnikar, Chao Zhang, Lakshmi Spandana Gudapati, Paulino Alvarez, Matthew E Deren, Nicolas S Piuzzi

**Affiliations:** 1 Cleveland Clinic, Department of Orthopedic Surgery, Cleveland, OH USA; 2 Case Western Reserve University School of Medicine, Cleveland, OH USA; 3 Cleveland Clinic, Heart, Vascular, and Thoracic Institute, Cleveland, OH USA; 4 Cleveland Clinic, Department of Biomedical Engineering, Cleveland, OH USA

**Keywords:** Heart failure, Total knee arthroplasty, Knee disability and osteoarthritis outcome score, Patient-reported outcome measures

## Abstract

**Purpose:**

Many heart failure (HF) patients undergo total knee arthroplasty (TKA), but their postoperative outcomes remain unclear. This study aimed to compare healthcare resource utilization and patient-reported outcome measures (PROMs) after TKA between patients with and without HF.

**Methods:**

A retrospective analysis of 12,491 TKA at our institution from 2016 to 2021, including 495 with HF. HF patients were stratified into three ejection fraction (EF) categories: preserved (≥ 50%, *n* = 374), mildly reduced (41–49%, *n* = 53), and reduced (≤ 40%, *n* = 68). Healthcare utilization metrics and 1-year mortality were compared. PROMs were assessed using the Knee Injury and Osteoarthritis Outcome Score for Pain (KOOS-Pain), Physical Function Shortform (KOOS-PS), and Joint Replacement (KOOS-JR) at baseline and 1-year postoperatively. Minimal clinically important difference (MCID) and patient-acceptable symptom state (PASS) thresholds were evaluated.

**Results:**

HF patients had significantly higher odds of prolonged hospital stay (OR 2.55, *p* < 0.001), non-home discharge (OR 2.17, *p* < 0.001), 90-day readmission (OR 2.02, *p* < 0.001), 90-day emergency department visits (OR 1.55, *p* = 0.002), and 1-year mortality (OR 3.53, *p* = 0.007). PROMs were similar between HF and non-HF patients at 1 year, though HF patients were more likely to achieve MCID for KOOS-PS (*p* = 0.021). Among EF subgroups, patients with mildly reduced EF had significantly higher 1-year KOOS-Pain (*p* = 0.024) and PASS achievement for pain (*p* = 0.043). EF did not predict 1-year outcomes.

**Conclusion:**

Despite increased healthcare utilization, HF patients undergoing TKA achieve similar improvements in pain and functionality as non-HF patients. HF severity was not associated with differential healthcare utilization, suggesting that risk stratification based on HF severity may not be necessary.

*Level of evidence *III.

**Supplementary Information:**

The online version contains supplementary material available at 10.1007/s00590-025-04549-1.

## Introduction

Heart failure (HF) is the third most common heart disease in the USA, and worldwide prevalence is estimated to be 64 million, with recent data showing that HF has increased steadily over the past 30 years worldwide [1, 2]. Moreover, the American Heart Association (AHA) has projected a relative increase in prevalence of 22.7% by 2030 [3], and this projection reflects an increase both in the USA and worldwide [4]. Heart failure can be classified according to left ventricular ejection fraction (LVEF) into: HF with preserved ejection fraction (EF) (HFpEF) is an EF ≥ 50%, HF with mildly reduced EF (HFmrEF) is an EF of 41–49%, and HF with reduced EF (HFrEF) is an EF ≤ 40% [5, 6].

Previous studies have described that individuals with osteoarthritis (OA) are twice as likely to experience HF compared with people without OA [7]. The relationship between these has been hypothesized to be complex and multifactorial, but as treatment and life expectancy for patients with HF continue to improve [8], the number of patients with HF that undergo TKA will likely increase. Previous observations have noted an increased risk of cardiac complications following TKA in patients with a history of heart disease and other risk factors, and patients with HF undergoing TKA have been found to be at higher risk for adverse perioperative outcomes and short-term complications compared to those without heart failure [9, 10]. Some of the complications that have been reported are longer hospital stays, readmission, wound dehiscence, and return to the operating room [9], but there is a paucity of information regarding patient-perceived outcomes based on HF severity following TKA. Due to the existing knowledge gap in PROMs for TKA patients with a diagnosis of HF, this study has two specific aims: 1) compare healthcare resource utilization, 1-year PROMs, and achievement of MCID and PASS thresholds at 1 year between patients with HF compared to patients without HF and 2) separate HF patients into the three HF categories of HFpEF, HFmrEF, and HFrEF and compare patients in these categories to determine any differences in healthcare resource utilization, 1-year PROMs, and achievement of MCID and PASS thresholds.

## Patients/methods

### Cohort and data curation

**Fig. 1:**
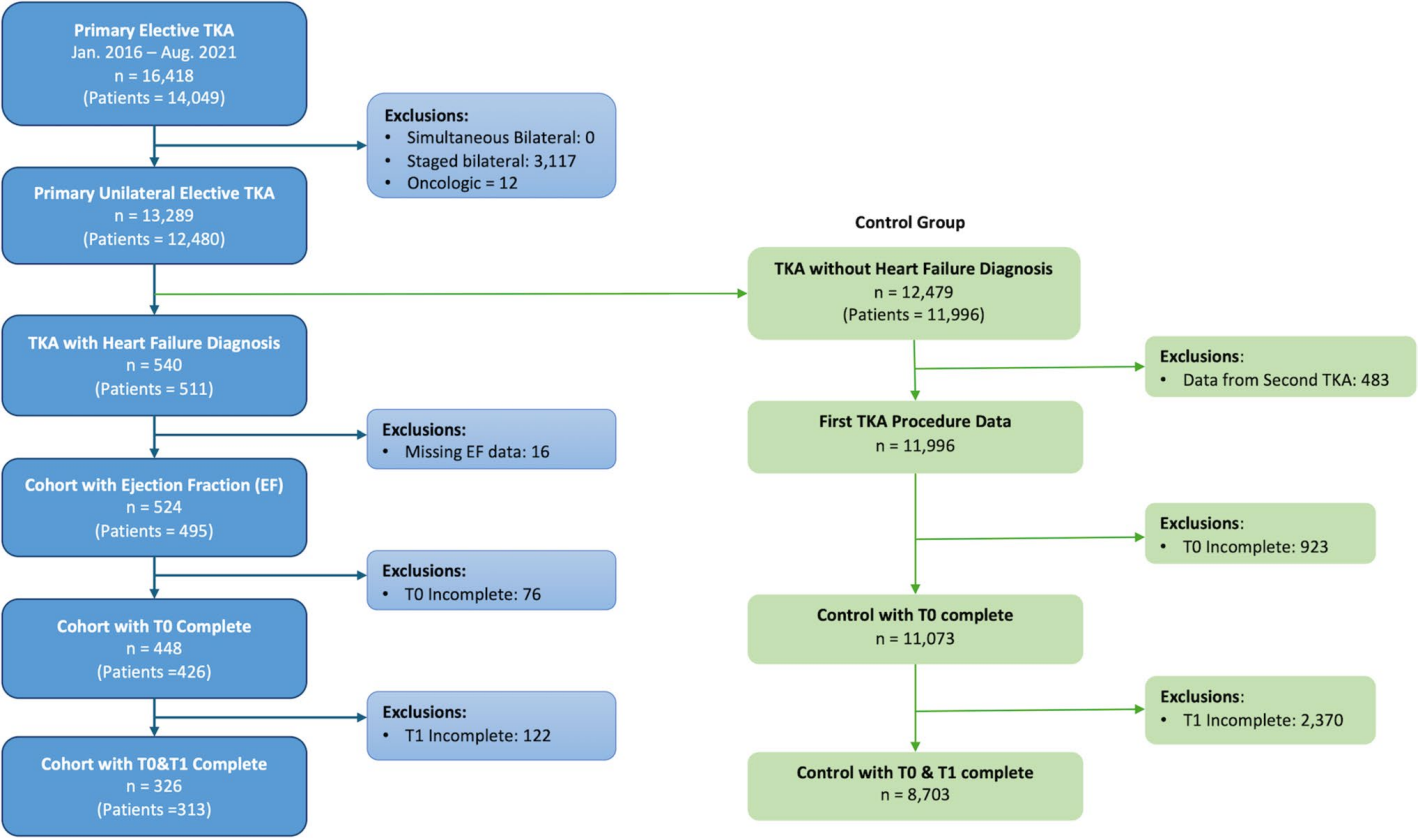
Strengthening the reporting of observational studies in epidemiology (STROBE) diagram

A total of 12,491 patients underwent primary, unilateral, elective TKA between January 1, 2016, and August 31, 2021, at a single academic tertiary center. Of these patients, 495 patients had a diagnosis of HF with available EF data. This resulted in the control cohort of 11,996 patients without a diagnosis of HF (Fig. 1). A validated institutional data collection system—Orthopaedic Minimal Data Set Episode of Care (OME)—was used to prospectively collect baseline and follow-up data [11–13]. Patients with HF were further stratified into ejection fraction (EF) categories, with 374 patients with HFpEF, 53 patients with HFmrEF, and 68 patients with HFrEF.

### Patient demographics

 When comparing patients without a diagnosis of HF to those with a diagnosis of HF, patients with HF were significantly older (*p* < 0.001); had a significantly greater body mass index (BMI) (*p* < 0.001), area deprivation index (ADI) (*p* < 0.001), and Charlson comorbidity index (CCI) (*p* < 0.001); and had significantly lower baseline KOOS-Pain (*p* < 0.001), KOOS-PS (*p* < 0.001), KOOS-JR (*p* < 0.001), and MCS scores (*p* = 0.001), among other differences (Table 1). When HF patients were divided into the three EF groups, differences in BMI (*p* = 0.028) and CCI (*p* = 0.042) remained among the groups, but no differences were observed for PROMs or other baseline demographics (Supplemental Table 1).

**Table 1:** Baseline demographics by heart failure diagnosis

Variable	Level	All (*n* = 12,491)	No HF (*n* = 11,996)	HF (*n* = 495)	*P*-value	*N*
Age		67.0 [60.0;73.0]	67.0 [60.0;73.0]	70.0 [64.0;77.0]	** < 0.001**	12,491
Sex	F	7538 (60.3%)	7260 (60.5%)	278 (56.2%)	0.058	12,491
	M	4953 (39.7%)	4736 (39.5%)	217 (43.8%)		
BMI		31.6 [27.5;36.3]	31.6 [27.5;36.2]	32.6 [28.4;38.3]	** < 0.001**	11,976
Education		14.0 [12.0;16.0]	14.0 [12.0;16.0]	12.0 [12.0;16.0]	** < 0.001**	11,497
ADI		56.0 [35.0;77.0]	55.0 [35.0;77.0]	62.0 [41.0;83.0]	** < 0.001**	12,025
Race	White	9979 (82.5%)	9601 (82.7%)	378 (77.3%)	**0.003**	12,098
	Non-white	2119 (17.5%)	2008 (17.3%)	111 (22.7%)		
Smoking	Never	6439 (56.0%)	6257 (56.5%)	182 (42.8%)	** < 0.001**	11,497
	Current	834 (7.25%)	800 (7.23%)	34 (8.00%)		
	Quit 0–6 m	385 (3.35%)	370 (3.34%)	15 (3.53%)		
	Quit 6 m +	3839 (33.4%)	3645 (32.9%)	194 (45.6%)		
CCI		1.00 [0.00;2.00]	0.00 [0.00;2.00]	2.00 [1.00;4.50]	** < 0.001**	12,332
Categorical CCI	0	6148 (49.9%)	6100 (51.5%)	48 (9.70%)	** < 0.001**	12,332
	1	2618 (21.2%)	2522 (21.3%)	96 (19.4%)		
	2	1706 (13.8%)	1600 (13.5%)	106 (21.4%)		
	3+	1860 (15.1%)	1615 (13.6%)	245 (49.5%)		
Insurance	Other	3974 (36.2%)	3898 (37.0%)	76 (17.0%)	** < 0.001**	10,990
	Medicaid or medicare	7016 (63.8%)	6644 (63.0%)	372 (83.0%)		
Baseline KOOS-pain		38.9 [30.6;50.0]	38.9 [30.6;50.0]	36.1 [25.0;47.2]	** < 0.001**	11,499
Baseline KOOS-PS		51.5 [38.0;58.0]	51.5 [38.0;58.0]	48.8 [33.4;56.0]	** < 0.001**	11,493
Baseline KOOS-JR		44.9 [36.9;52.5]	44.9 [36.9;52.5]	42.3 [34.2;50.0]	** < 0.001**	10,273
Baseline MCS		51.5 [41.5;60.3]	51.6 [41.7;60.4]	50.1 [38.6;58.3]	**0.001**	11,499
Phenotype	P + PS + MCS +	2870 (25.0%)	2793 (25.2%)	77 (18.1%)	**0.008**	11,493
	P-PS + MCS +	699 (6.08%)	676 (6.11%)	23 (5.41%)		
	P + PS-MCS +	603 (5.25%)	574 (5.19%)	29 (6.82%)		
	P + PS + MCS-	1624 (14.1%)	1570 (14.2%)	54 (12.7%)		
	P + PS-MCS-	544 (4.73%)	525 (4.74%)	19 (4.47%)		
	P-PS-MCS +	1576 (13.7%)	1511 (13.7%)	65 (15.3%)		
	P-PS + MCS-	667 (5.80%)	643 (5.81%)	24 (5.65%)		
	P-PS-MCS-	2910 (25.3%)	2776 (25.1%)	134 (31.5%)		

*BMI* body mass index, *ADI* area deprivation index, *CCI* Charlson comorbidity index, *KOOS* Knee disability and osteoarthritis outcome score, *PS* physical function shortform, *JR* joint replacement, *MCS* mental component summary

Bold numbers indicate significant values

Continuous variables presented as Median [IQR]. Categorical variables presented as *N* (%)

### Outcomes of interest

Demographic characteristics were recorded including sex, age, race, body mass index (BMI), area deprivation index (ADI), which is a scaled measure with larger scores indicating residence in a neighborhood of greater socioeconomic deprivation and baseline comorbidities. Healthcare resource utilization, including LOS ≥ 3 days, DD, 90-day hospital readmission, 90-day ED visit, 1-year reoperation, and mortality up to 1 year, was recorded. The PROMs included the Knee disability and Osteoarthritis Outcome Score for Pain (KOOS-Pain), KOOS for Joint Replacement (KOOS-JR), KOOS Physical Function Shortform (KOOS-PS), and the Veterans RAND 12-Item Health Survey Mental Component Score (MCS) [14–16]. PROMs were collected just before surgery (baseline) and at 1-year follow-up and were recorded in the Research Electronic Data Capture (REDCap) database. The MCID reflects an estimate of the minimum PROM change/improvement that patients can perceive. MCID was calculated through distribution-based methods [17–19], and the values used were 7.99 for KOOS-Pain, 8.04 for KOOS-PS, and 6.76 for KOOS-JR. PASS values represent thresholds that indicated optimal patient satisfaction and were estimated using an anchor-based approach [20]. The PASS values were ≥ 77.7 for KOOS-Pain and ≥ 70.3 for both KOOS-PS and KOOS-JR [21].

### Statistical analysis

Continuous variables were presented using mean and standard deviations (SD), or medians and interquartile ranges (IQRs), when appropriate. Categorical variables were displayed using counts and percentages.

All models were adjusted for the following clinical and demographic covariates: age, sex, BMI, race, education, smoking status, ADI, CCI, insurance type, PROM phenotype, and baseline KOOS-Pain, PS, JR, and MCS. After adjusting for demographic factors, multivariable linear regression was conducted to examine the relationship between ejection fraction and 1-year PROMs (Pain, PS, JR at 1-year). Multivariable logistic regression was employed to assess the relationship between EF and PASS threshold as well as healthcare outcomes. For all MCID outcomes, reoperation, and mortality, a Chi-squared test was performed to assess differences among the three EF groups, given the limited event size.

To evaluate the association between HF and outcomes, we utilized propensity matching. This was necessary because there were only 495 patients with a diagnosis of HF, compared to 11,996 patients without HF. To balance the distribution of confounders and achieve balanced distributions across different levels of HF, propensity score matching was employed on covariates selected by physicians based on their clinical knowledge. To address missing values, multiple imputation methods with chained equations (MICE) were utilized to impute missing data for both analysis cohorts. Following the generation of 20 multiply imputed datasets, the within approach was then used to conduct matching with the multiply imputed data. After assessing the balance in the matched datasets and achieving balance, the effects of HF and their standard errors were estimated within each imputed dataset and then pooled to obtain a single set of coefficients and standard errors.

**Fig. 2:**
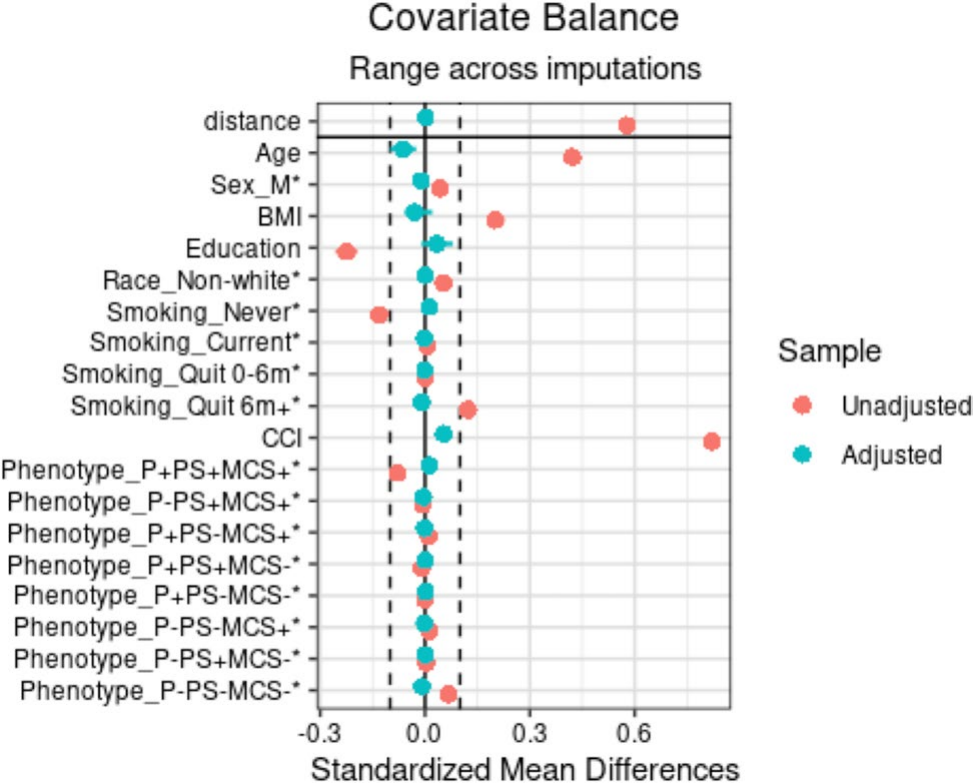
Standardized mean differences for covariates

The initial attempt at matching involved 1:3 nearest-neighbor propensity score matching without replacement, through logistic regression on the covariates for both cohorts. This matching method resulted in relatively acceptable balance. To further enhance the balance between the groups, alternative link functions (such as probit and linear logit links) and matching methods (including optimal and exact matching for one of the covariates, phenotype) were implemented. Ultimately, the original matching method (nearest neighbor, logit link with a 1:3 ratio) was chosen for cohorts with healthcare utilization outcome data and for the patients with 1-year PROM data. All standardized mean differences for covariates were below 0.1, indicating satisfactory balance (Fig. 2).

To estimate the exposure effect of heart failure and its standard error, we accounted for the weights and clusters in each imputed dataset. We estimated the exposure effect of HF and its confidence interval in each dataset and then pooled the results to derive the final coefficients and standard errors. Analysis was conducted using R (Austria, version 4.3.1), and the MICE, MatchThem, Cobalt, and Survey packages were utilized. All tests were two-sided, assuming an alpha level of 0.05.

## Results

### Healthcare resource utilization

Patients with a diagnosis of HF more often experienced prolonged LOS (*p* < 0.001), non-home DD (*p* < 0.001), 90-day hospital readmission (*p* < 0.001), and 90-day ED visit (*p* < 0.001) (Table 2). Patients with HF more often experienced mortality within 1 year of TKA, with 16 of the 495 (3.23%) HF patients dying within a year of their procedure, compared to 60 of the 11,996 (0.50%) non-HF patients (*p* < 0.001) (Table 2). Furthermore, after propensity matching, HF functioned as a predictor for prolonged LOS (odds ratio (OR): 2.55, 95% confidence interval (CI): 1.98–3.29, *p* < 0.001), non-home DD (OR: 2.17, 95% CI: 1.61–2.91, *p* < 0.001), 90-day hospital readmission (OR: 2.02, 95% CI: 1.49–2.72, *p* < 0.001), 90-day ED visit (OD: 1.55, 95% CI: 1.18–2.04, *p* = 0.002), and 1-year mortality (OR: 3.53, 95% CI: 1.43–8.73, *p* = 0.007) (Supplemental Table 2).

**Table 2:** Healthcare resource utilization outcomes by heart failure diagnosis

Variable	Level	All (* n* = 12,491)	No (* n* = 11,996)	Yes (* n* = 495)	*P*-value	*N*
LOS ≥ 3		1922 (15.5%)	1729 (14.5%)	193 (39.0%)	** < 0.001**	12,431
DD	Home/home health care	11,220 (90.3%)	10,863 (91.0%)	357 (72.1%)	** < 0.001**	12,426
	Non-home	1206 (9.71%)	1068 (8.95%)	138 (27.9%)		
90-day readmission		1047 (8.42%)	945 (7.92%)	102 (20.6%)	** < 0.001**	12,431
90-day ED visit		1888 (15.1%)	1758 (14.7%)	130 (26.3%)	** < 0.001**	12,491
1-year reoperation		583 (4.67%)	563 (4.69%)	20 (4.04%)	0.571	12,491
1-year mortality		76 (0.61%)	60 (0.50%)	16 (3.23%)	** < 0.001**	12,491

*LOS* length of stay, *DD* discharge disposition, *ED* emergency department

Bold numbers indicate significant values

Categorical variables presented as *N* (%). Bold numbers indicate significant values

When comparing healthcare resource utilization outcomes for HF patients stratified into the three EF categories, no associations were found among the groups (Supplemental Table 3). After adjusting other variables in the logistic regression, EF did not function as a predictor for LOS, DD, readmission, or ED visit outcomes (Supplemental Table 4). Given the overall low sample size and the low number of events for 1-year reoperation and mortality, these outcomes were not included in the regression model.

### Changes in patient-reported outcome measures

There was no significant difference in 1-year postoperative TKA PROMs or achievement of PASS thresholds for any KOOS outcomes when comparing HF and non-HF patients. For the achievement of MCID, a significantly greater proportion of HF patients achieved MCID for KOOS-PS compared to non-HF patients (*p* = 0.021) (Table 3). After matching, HF did not impact achievement of MCID and PASS thresholds (Supplemental Table 2). Overall, > 85% of all patients achieved MCID for all KOOS outcomes. Upon further stratification into the three EF categories, HF patients with mildly reduced EF had significantly higher 1-year KOOS-Pain score (*p* = 0.024) and achievement of PASS threshold for KOOS-Pain (*p* = 0.043) (Supplemental Table 5).

**Table 3:** 1-year outcomes by heart failure diagnosis

Variable	Level	All (* n* = 8,944)	No (* n* = 8,634)	Yes (* n* = 310)	*P*-value	*N*
KOOS-pain		86.1 [72.2;97.2]	86.1 [72.2;97.2]	88.9 [75.0;97.2]	0.237	8944
KOOS-PS		75.1 [64.7;85.2]	75.1 [64.7;85.2]	75.1 [64.7;85.2]	0.631	8532
KOOS-JR		76.3 [66.0;92.0]	76.3 [66.0;92.0]	76.3 [66.0;92.0]	0.558	7455
MCID KOOS-pain	Achieved	8370 (93.6%)	8075 (93.5%)	295 (95.2%)	0.300	8944
	Treatment failure	574 (6.42%)	559 (6.47%)	15 (4.84%)		
MCID KOOS-PS	Achieved	7276 (85.3%)	7017 (85.2%)	259 (90.2%)	**0.021**	8527
	Treatment failure	1251 (14.7%)	1223 (14.8%)	28 (9.76%)		
MCID KOOS-JR	Achieved	6831 (91.7%)	6599 (91.6%)	232 (94.3%)	0.159	7452
	Treatment failure	621 (8.33%)	607 (8.42%)	14 (5.69%)		
PASS threshold for pain	Achieved	6173 (69.0%)	5957 (69.0%)	216 (69.7%)	0.847	8944
	Treatment failure	2771 (31.0%)	2677 (31.0%)	94 (30.3%)		
PASS threshold for PS	Achieved	5559 (65.2%)	5381 (65.3%)	178 (62.0%)	0.284	8532
	Treatment failure	2973 (34.8%)	2864 (34.7%)	109 (38.0%)		
PASS threshold for JR	Achieved	4972 (66.7%)	4803 (66.6%)	169 (68.7%)	0.542	7455
	Treatment failure	2483 (33.3%)	2406 (33.4%)	77 (31.3%)		

*KOOS* knee disability and osteoarthritis outcome score, *PS* physical function shortform, *JR* joint replacement, *MCID* minimal clinically important difference, *PASS* patient acceptable symptom state

Bold numbers indicate a significant value

Continuous variables presented as Median [IQR]. Categorical variables presented as N (%)

With regard to regression analysis, EF did not serve as a significant predictor of 1-year KOOS scores (Supplemental Table 6) or achievement of PASS for any KOOS outcome (Supplemental Table 7). Achievement of MCID was not included in the regression analysis given the low number of HF patients failing to achieve MCID for all KOOS outcomes.

## Discussion

The rising prevalence of heart failure (HF) presents a significant challenge for patients undergoing total knee arthroplasty (TKA), given the increased risk of complications and healthcare resource utilization. In this study, we aimed to compare healthcare resource utilization and 1-year patient-reported outcome measures (PROMs) between patients with and without HF, and further stratified HF patients by ejection fraction (EF) categories to explore differences within the HF cohort. Our findings indicate that while patients with HF experience greater healthcare utilization post-TKA, their 1-year PROMs are comparable to those without HF. Patients undergoing TKA with a diagnosis of HF had greater BMI, ADI, and CCI, which is consistent with the current literature [22–25]. Differences in BMI and CCI were also present when the HF patients were stratified into the different EF categories. Patients with HF had significantly lower baseline KOOS-Pain, KOOS-PS, KOOS-JR, and MCS scores compared to non-HF patients. However, when HF patients were stratified by EF category, no significant differences in baseline PROMs were observed between the EF groups. This finding suggests that although baseline KOOS scores may be worse for HF patients, the degree of HF, as measured by EF, does not appear to have a correlation with baseline PROMs. To the best of our knowledge, there are currently no studies analyzing improvement in PROM scores by severity of HF in patients who underwent TKA.

Regarding postoperative healthcare resource utilization outcomes, patients with a diagnosis of HF more often experienced prolonged LOS, non-home DD, 90-day hospital readmission, 90-day ED visit, and 1-year mortality compared to patients without a diagnosis of HF. However, there were no associations between healthcare utilization and the degree of HF, as measured by EF, thus suggesting a lack of a dose–response relationship between HF severity and healthcare resource utilization outcomes. An association between HF diagnosis and prolonged LOS was highlighted in a retrospective study where Gholson et al. [26] analyzed 92,266 patients and found that HF was associated with significantly increased LOS by 1.46 days in the total joint arthroplasty population (*p* < 0.05) [26]. A study that analyzed patients with congestive HF also found that patients with preexisting HF were at increased risk of a prolonged LOS following TKA when compared to the control cohort (3.54 days vs 1.94 days, *p* < 0.001) and that HF patients were more likely to experience non-home DD (*p* < 0.001) [27]. Curtis et al. [9] analyzed 251 patients with HF who underwent TKA and found that patients with HF had a significantly greater average in-hospital LOS compared to patients without HF (4.1 ± 3.3 vs. 3.2 ± 3.8 days; *p* < 0.001) and higher rates of non-home DD (*p* < 0.001), readmission (*p* < 0.001), and reoperation (*p* < 0.001) compared to non-HF patients. In addition, Curtis et al. [9] found that HF patients were more likely to have reoperation (OR 2.00, 95% CI 1.01–3.94, *p* = 0.046) and readmission (OR 1.88, 95% CI 1.21–2.94, *p* = 0.005) following TKA compared to controls. Overall, the association between HF and readmission after TKA has been well established through several studies [28, 29], with one study’s results showing congestive HF to be the greatest predictor of readmission (OR 1.59, 95% CI 1.43–1.78, *p* < 0.001), with a median cost for each readmission of $6753 ± 175 (IQR) and a mean cost for readmission of $10,465 ± 15,257 (SD), which accounted for roughly 36% of the overall total in-patient cost in the first 30 days following TKA [30]. Similarly, Young et al. [31] found that patients with HF had a longer LOS (5 vs. 4 days, *p* < 0.0001) and a higher rate of developing 90-day medical complications (49.22% vs. 7.45%, OR: 6.89, 95% CI: 6.82–6.96, *p* < 0.0001) after TKA, which overall resulted in greater day of surgery ($12,362 vs. $11,873, *p* < 0.0001) and ninety-day episode of care costs ($16,373 vs. $14,479, *p* < 0.0001) compared to a matched control cohort. With these factors in mind, our findings provide evidence that HF should be considered a high-impact comorbidity in surgical planning and payment models including bundled payments and outlier cost reimbursements.

Overall, patients with HF who undergo TKA show an increased rate of healthcare resource utilization compared to patients without HF. This is important because previous studies have explored the rate of outlier payments for TKA procedures and discussed the potential long-term implications for hospital budgets. Outlier payments are funds provided by the Centers for Medicare and Medicaid Services (CMS) for arthroplasty procedures that are exceptionally complex or involve high costs [32]. A study by Li et al. [32] that explored Medicare payments for TKA discussed how CMS has recently tightened restrictions and dramatically reduced the amount of outlier payments over time with concerns that some hospitals may be misusing these resources. In their study, Li et al. [32] found that for both primary and revision TKAs, outlier payments were associated with TKA procedures of patients that had concurrent HF. This raises concern as the stricter payment policies could create financial disincentives for hospitals to perform TKA on appropriately selected HF patients. Moreover, this issue becomes more critical as the number of HF patients undergoing TKAs is projected to continue increasing in the future.

Interestingly, Curtis et al. [9] results did not show an association between a diagnosis of HF and mortality following TKA, results that conflict with the current study. However, Okpara et al. [27] results do align with the current study as they found that patients with HF were more likely to experience mortality following TKA compared to the control cohort (*p* < 0.001). Organizations with mortality data available for patients with HF who have already undergone TKA should aim to publish results and support or refute the current findings.

No significant differences were found for 1-year KOOS-Pain, KOOS-JR, and KOOS-PS. Similarly, there was no significant difference in the achievement of the PASS thresholds for any KOOS outcome at 1-year postoperative follow-up. However, there was a significant difference in achievement of MCID for KOOS-PS, where, counterintuitively, a higher proportion of HF patients achieved MCID for KOOS-PS compared to non-HF patients. Given the poor postoperative healthcare resource utilization outcomes among HF patients, it is interesting that HF patients have PROM scores that are similar to non-HF patients, with a greater proportion of HF patients achieving MCID for KOOS-PS. This may be due to the fact that HF patients report worse baseline KOOS scores, thus leaving more room for perceived improvement for these patients. Similar results were seen when the HF patients were stratified into EF categories, with the only differences among the EF categories being for KOOS-Pain and achievement of the PASS threshold for KOOS-Pain. Specifically, patients with a greater EF demonstrated worse pain at 1 year postoperatively and a lower rate of achievement of the PASS threshold for KOOS-Pain. It is possible that patients with lower EF tend to have lower activity levels, thus resulting in greater pain improvement. On the contrary, patients with a greater EF are thus able to have higher activity levels, resulting in greater postoperative pain and lower rates of achievement of PASS for KOOS-Pain. Altogether, the lack of association between EF subgroups and PROM threshold achievement or healthcare utilization despite differences based on the presence of HF suggests that EF alone may not be sufficient for risk stratification in TKA patients, calling for broader risk stratification tools including functional and biochemical markers. However, to the best of our knowledge, there are currently no other studies analyzing PROMs for HF patients who underwent TKA.

This study is not without limitations. First, there are several confounding factors associated with HF, such as patient race and BMI [33, 34]. While our results are independent of race, future studies may need to stratify patients by racial and/or ethnic background to provide a more comprehensive evaluation [35–40]. Analyzing postoperative medical complications was not done in the current study. Previous research has shown that HF patients undergoing TKA have higher rates of deep vein thrombosis, pneumonia, cerebrovascular accident, myocardial infarction, acute renal failure, sepsis, urinary tract infection, risk of revision TKA, periprosthetic joint infection, aseptic loosening, and dislocation when compared to non-HF patients [9, 41–43]. It is crucial to take these surgical consequences into account when deciding whether a patient qualifies for TKA. Yet, because these postoperative complications would likely result in greater usage of hospital resources, they further underscore the need for improved compensation for HF TKA patients. Second, although selection bias measures were employed, this single-center setting and observational nature of our study limit generalizability. Furthermore, the HF group in our cohort remained comparatively small to our control group (*n* = 495 vs. 11,996), which does limit the statistical power to detect meaningful differences within HF categories. Nevertheless, this study represents the largest HF cohort to date examining perioperative healthcare utilization and validated PROM thresholds following TKA. Third, it is possible that HF patients with more severe frailty were not selected for TKA, introducing survivorship and/or referral bias. Although we adjusted for baseline PROM phenotype and patient comorbidities in our multivariate analyses, there remains a need for broader evaluation on the impact of functional recovery and quality of life following surgery among patients with HF. In addition, while our study stratified HF patients based on EF, stratification by alternative dimensions including New York Heart Association (NYHA) classification, diastolic dysfunction, natriuretic peptide levels, or exercise tolerance may yield different results. Another limitation is the exclusion of postoperative complication data, including deep vein thrombosis, myocardial infarction, and sepsis, which may have provided a more complete risk profile. Although this datum was unavailable to the authors, future studies can benefit from characterizing such factors in their cohort as they may help shed light on the underlying explanation for higher rates of healthcare utilization. Future work can provide additional value by utilizing a multicenter design of a diverse patient population, stratification of HF by the NYHA classification or functional or biochemical markers, prospectively evaluating longer-term complication rates, and performing economic analyses to quantify the impact downstream costs in HF patients undergoing TKA.

## Conclusion

Patients with HF undergoing TKA demonstrated higher likelihood of prolonged LOS, non-home discharge, 90-day hospital readmission, 90-day ED visit, and 1-year mortality. However, there was no association between HF EF category and any healthcare resource utilization outcome, thus highlighting the possibility that risk stratification for TKA by HF severity may not be warranted. Furthermore, there was no significant difference in 1-year PROMs, regardless of HF presence or absence, with > 85% of all patients achieving the MCID for all KOOS outcomes. Thus, after patient-provider discussion about a greater risk for postoperative healthcare resource utilization and mortality, if patients are deemed eligible for operation, they can expect excellent outcomes in pain and functionality 1-year following TKA. Given the promising postoperative PROMs, it is imperative that hospitals are adequately compensated when performing TKA on high resource-utilizing patients such as those with HF.

## Supplementary Information

Below is the link to the electronic supplementary material.

Supplementary file1 (DOCX 32 kb)

## Data Availability

No datasets were generated or analysed during the current study.
