# Supplementary material for: Heart failure patients demonstrate excellent 1-year outcomes after total knee arthroplasty despite high healthcare utilization
Source: Eur J Orthop Surg Traumatol. 2025 Oct 9;35(1):424. doi: 10.1007/s00590-025-04549-1 (PMC12511152; doi:10.1007/s00590-025-04549-1)
Supplement: Supplementary file 1 — Supplementary file1 (DOCX 32 kb) [file 590_2025_4549_MOESM1_ESM.docx]

**Supplementary Material**

| **Table 1.** Baseline Demographics by Heart Failure Ejection Fraction Category | | | | | | | |
| --- | --- | --- | --- | --- | --- | --- | --- |
| **Variable** | **Level** | **All (n=495)** | **HFrEF (n=68)** | **HFmrEF (n=53)** | **HFpEF (n=374)** | **P-value** | **N** |
| **Age** |  | 70.2 (9.17) | 69.5 (9.41) | 71.6 (8.87) | 70.2 (9.18) | 0.453 | 495 |
| **Sex** | F | 278 (56.2%) | 22 (32.4%) | 22 (41.5%) | 234 (62.6%) | **<0.001** | 495 |
|  | M | 217 (43.8%) | 46 (67.6%) | 31 (58.5%) | 140 (37.4%) |  |  |
| **BMI** |  | 32.6 [28.4;38.3] | 32.0 [27.3;36.5] | 31.0 [27.2;35.7] | 33.2 [28.9;38.9] | **0.028** | 457 |
| **Education** |  | 12.0 [12.0;16.0] | 13.0 [12.0;16.0] | 12.0 [12.0;15.2] | 12.0 [12.0;16.0] | 0.415 | 425 |
| **ADI** |  | 62.0 [41.0;83.0] | 54.5 [33.8;75.8] | 59.5 [42.2;79.2] | 64.5 [42.8;84.2] | 0.149 | 474 |
| **Race** | White | 378 (77.3%) | 56 (84.8%) | 41 (80.4%) | 281 (75.5%) | 0.214 | 489 |
|  | Non-white | 111 (22.7%) | 10 (15.2%) | 10 (19.6%) | 91 (24.5%) |  |  |
| **Smoking** | Never | 182 (42.8%) | 31 (55.4%) | 18 (37.5%) | 133 (41.4%) | 0.590 | 425 |
|  | Current | 34 (8.00%) | 3 (5.36%) | 4 (8.33%) | 27 (8.41%) |  |  |
|  | Quit 0-6m | 15 (3.53%) | 1 (1.79%) | 1 (2.08%) | 13 (4.05%) |  |  |
|  | Quit 6m+ | 194 (45.6%) | 21 (37.5%) | 25 (52.1%) | 148 (46.1%) |  |  |
| **CCI** |  | 2.00 [1.00;4.50] | 3.00 [2.00;5.00] | 3.00 [1.00;4.00] | 2.00 [1.00;4.00] | **0.042** | 495 |
| **Categorical CCI** | 0 | 48 (9.70%) | 1 (1.47%) | 4 (7.55%) | 43 (11.5%) | **0.033** | 495 |
|  | 1 | 96 (19.4%) | 9 (13.2%) | 15 (28.3%) | 72 (19.3%) |  |  |
|  | 2 | 106 (21.4%) | 18 (26.5%) | 7 (13.2%) | 81 (21.7%) |  |  |
|  | 3+ | 245 (49.5%) | 40 (58.8%) | 27 (50.9%) | 178 (47.6%) |  |  |
| **Insurance** | Other | 76 (17.0%) | 10 (15.2%) | 10 (21.7%) | 56 (16.7%) | 0.631 | 448 |
|  | Medicaid or Medicare | 372 (83.0%) | 56 (84.8%) | 36 (78.3%) | 280 (83.3%) |  |  |
| **Baseline KOOS-Pain** |  | 36.1 [25.0;47.2] | 40.3 [27.8;52.8] | 37.5 [27.8;52.8] | 36.1 [25.0;47.2] | 0.174 | 425 |
| **Baseline KOOS-PS** |  | 48.8 [33.4;56.0] | 51.5 [36.9;59.7] | 48.8 [38.0;56.0] | 45.6 [33.4;56.0] | 0.185 | 425 |
| **Baseline KOOS-JR** |  | 42.3 [34.2;50.0] | 44.9 [34.2;52.5] | 42.3 [34.2;52.5] | 42.3 [34.2;47.5] | 0.251 | 384 |
| **Baseline MCS** |  | 50.1 [38.6;58.3] | 51.8 [37.4;56.8] | 51.3 [42.4;59.1] | 48.9 [38.3;58.4] | 0.391 | 425 |
| Continuous variables presented as Median [IQR]. Categorical variables presented as N (%). Bold numbers indicate significant values.  HFrEF – heart failure with reduced ejection fraction; HFmrEF – heart failure with mildly reduced ejection fraction; HFpEF – heart failure with preserved ejection fraction; BMI – body mass index; ADI – area deprivation index; CCI – Charlson comorbidity index; KOOS – knee disability and osteoarthritis outcome score; PS – physical function shortform; JR – joint replacement; MCS – mental component summary. | | | | | | | |

| **Table 2.** Estimated Treatment Effect of Heart Failure Diagnosis | | |
| --- | --- | --- |
| **Model Names** | **Heart Failure (Yes v. No)   Odds Ratio [95% CI]** | **P-value** |
| **Pain MCID (Failure)** | 0.74 (0.39, 1.39) | 0.341 |
| **PS MCID (Failure)** | 0.7 (0.45, 1.09) | 0.113 |
| **JR MCID (Failure)** | 0.77 (0.43, 1.38) | 0.375 |
| **Pain PASS (Failure)** | 0.88 (0.64, 1.21) | 0.442 |
| **PS PASS (Failure)** | 0.99 (0.73, 1.34) | 0.949 |
| **JR PASS (Failure)** | 0.85 (0.62, 1.17) | 0.311 |
| **LOS ≥3** | 2.55 (1.98, 3.29) | **<0.001** |
| **DD** | 2.17 (1.61, 2.91) | **<0.001** |
| **Readmission** | 2.02 (1.49, 2.72) | **<0.001** |
| **ED Visit** | 1.55 (1.18, 2.04) | **0.002** |
| **Reoperation** | 0.92 (0.52, 1.61) | 0.764 |
| **Mortality** | 3.53 (1.43, 8.73) | **0.007** |
| Bold numbers indicate significant values. CI – confidence interval; MCID – minimal clinically important difference; PASS – patient acceptable symptom state; PS – physical function shortform; JR – joint replacement; LOS – length of stay; DD – discharge disposition; ED – emergency department | | |

| **Table 3.** Health Care Outcomes by Heart Failure Ejection Fraction Category | | | | | | | |
| --- | --- | --- | --- | --- | --- | --- | --- |
| **Variable** | **Level** | **All (n=495)** | **HFrEF (n=68)** | **HFmrEF (n=53)** | **HFpEF (n=374)** | **P-value** | **N** |
| **LOS >=3** |  | 193 (39.0%) | 30 (44.1%) | 15 (28.3%) | 148 (39.6%) | 0.187 | 495 |
| **DD** | Home/home health care | 357 (72.1%) | 47 (69.1%) | 39 (73.6%) | 271 (72.5%) | 0.826 | 495 |
|  | Non-home | 138 (27.9%) | 21 (30.9%) | 14 (26.4%) | 103 (27.5%) |  |  |
| **90-day Readmission** |  | 102 (20.6%) | 20 (29.4%) | 11 (20.8%) | 71 (19.0%) | 0.148 | 495 |
| **90-day ED visit** |  | 130 (26.3%) | 23 (33.8%) | 14 (26.4%) | 93 (24.9%) | 0.304 | 495 |
| **1-year Reoperation** |  | 20 (4.04%) | 5 (7.35%) | 4 (7.55%) | 11 (2.94%) | 0.069 | 495 |
| **1-year Mortality** |  | 16 (3.23%) | 4 (5.88%) | 2 (3.77%) | 10 (2.67%) | 0.395 | 495 |
| Categorical variables presented as N (%). Bold numbers indicate significant values.  HFrEF – heart failure with reduced ejection fraction; HFmrEF – heart failure with mildly reduced ejection fraction; HFpEF – heart failure with preserved ejection fraction; LOS – length of stay; DD – discharge disposition; ED – emergency department | | | | | | | |

| **Table 4.** Heart Failure Category as a Predictor for Healthcare Resource Utilization Outcomes | | | | | | | | |
| --- | --- | --- | --- | --- | --- | --- | --- | --- |
|  | **LOS ≥3** | | **Non-home DD** | | **Readmission** | | **ED Visit** | |
| Predictors | *Estimates  [95% CI]* | *P-value* | *Estimates  [95% CI]* | *P-value* | *Estimates  [95% CI]* | *P-value* | *Estimates  [95% CI]* | *P-value* |
| EF (HFmrEF v. HFrEF) | -0.15 (-0.33 – 0.02) | 0.082 | -0.06 (-0.22 – 0.10) | 0.443 | -0.10 (-0.24 – 0.05) | 0.194 | -0.09 (-0.25 – 0.07) | 0.268 |
| EF (HFpER v. HFrEF) | -0.07 (-0.20 – 0.06) | 0.314 | -0.06 (-0.18 – 0.06) | 0.353 | -0.09 (-0.20 – 0.01) | 0.087 | -0.09 (-0.21 – 0.03) | 0.140 |
| Observations | 495 | | 495 | | 495 | | 495 | |
| R^2^ | 0.078 | | 0.059 | | 0.044 | | 0.038 | |

| **Table 5.** 1-year Outcomes by Heart Failure Ejection Fraction Category | | | | | | | |
| --- | --- | --- | --- | --- | --- | --- | --- |
| **Variable** | **Level** | **All (n=310)** | **HFrEF (n=38)** | **HFmrEF (n=33)** | **HFpEF (n=239)** | **P-value** | **N** |
| **KOOS-Pain** |  | 88.9 [75.0;97.2] | 91.7 [80.7;99.3] | 93.8 [83.3;100] | 86.1 [72.2;97.2] | **0.024** | 310 |
| **KOOS-PS** |  | 75.1 [64.7;85.2] | 81.4 [68.2;89.5] | 73.8 [67.3;85.2] | 72.5 [63.0;85.2] | 0.226 | 287 |
| **KOOS-JR** |  | 76.3 [66.0;92.0] | 84.6 [70.7;92.0] | 79.9 [70.7;92.0] | 73.3 [63.8;92.0] | 0.113 | 246 |
| **MCID KOOS-Pain** | Achieved | 295 (95.2%) | 36 (94.7%) | 32 (97.0%) | 227 (95.0%) | 0.924 | 310 |
|  | Treatment Failure | 15 (4.84%) | 2 (5.26%) | 1 (3.03%) | 12 (5.02%) |  |  |
| **MCID KOOS-PS** | Achieved | 259 (90.2%) | 34 (91.9%) | 24 (85.7%) | 201 (90.5%) | 0.727 | 287 |
|  | Treatment Failure | 28 (9.76%) | 3 (8.11%) | 4 (14.3%) | 21 (9.46%) |  |  |
| **MCID KOOS-JR** | Achieved | 232 (94.3%) | 32 (97.0%) | 24 (100%) | 176 (93.1%) | 0.396 | 246 |
|  | Treatment Failure | 14 (5.69%) | 1 (3.03%) | 0 (0.00%) | 13 (6.88%) |  |  |
| **PASS Threshold for Pain** | Achieved | 216 (69.7%) | 31 (81.6%) | 27 (81.8%) | 158 (66.1%) | **0.043** | 310 |
|  | Treatment Failure | 94 (30.3%) | 7 (18.4%) | 6 (18.2%) | 81 (33.9%) |  |  |
| **PASS Threshold for PS** | Achieved | 178 (62.0%) | 25 (67.6%) | 19 (67.9%) | 134 (60.4%) | 0.563 | 287 |
|  | Treatment Failure | 109 (38.0%) | 12 (32.4%) | 9 (32.1%) | 88 (39.6%) |  |  |
| **PASS Threshold for JR** | Achieved | 169 (68.7%) | 26 (78.8%) | 19 (79.2%) | 124 (65.6%) | 0.163 | 246 |
|  | Treatment Failure | 77 (31.3%) | 7 (21.2%) | 5 (20.8%) | 65 (34.4%) |  |  |
| Continuous variables presented as Median [IQR]. Categorical variables presented as N (column %). Bold numbers indicate significant values. HFrEF – heart failure with reduced ejection fraction; HFmrEF – heart failure with mildly reduced ejection fraction; HFpEF – heart failure with preserved ejection fraction; KOOS – knee disability and osteoarthritis outcome score; PS – physical function shortform; JR – joint replacement; MCID – minimal clinically important difference; PASS – patient acceptable symptom state. | | | | | | | |

| **Table 6.** Heart Failure Category as a Predictor for 1-Year PROMs | | | | | | |
| --- | --- | --- | --- | --- | --- | --- |
|  | **KOOS-Pain** | | **KOOS-PS** | | **KOOS-JR** | |
| Predictors | *Estimates  [95% CI]* | *P-value* | *Estimates  [95% CI]* | *P-value* | *Estimates  [95% CI]* | *P-value* |
| EF (HFmrEF vs. HFrEF) | 0.87 (-7.12 – 8.86) | 0.830 | -0.24 (-7.50 – 7.01) | 0.948 | 0.18 (-7.43 – 7.80) | 0.962 |
| EF (HFpER vs. HFrEF) | -4.70 (-10.85 – 1.46) | 0.135 | -2.17 (-7.74 – 3.41) | 0.445 | -1.94 (-7.77 – 3.90) | 0.514 |
| Observations | 312 | | 312 | | 312 | |
| R^2^ | 0.238 | | 0.211 | | 0.228 | |
| PROMs – patient-reported outcome measures; KOOS – knee disability and osteoarthritis outcome score; PS – physical function shortform; JR – joint replacement; CI – confidence interval; EF – ejection fraction; HFrEF – heart failure with reduced ejection fraction; HFmrEF – heart failure with mildly reduced ejection fraction; HFpEF – heart failure with preserved ejection fraction. | | | | | | |

| **Table 7.** Heart Failure Category as a Predictor for Failure to Reach PASS Thresholds for KOOS | | | | | | |
| --- | --- | --- | --- | --- | --- | --- |
|  | **PASS Pain (Treatment Failure)** | | **PASS PS (Treatment Failure)** | | **PASS JR (Treatment Failure)** | |
| Predictors | *Estimates  [95% CI]* | *P-value* | *Estimates  [95% CI]* | *P-value* | *Estimates  [95% CI]* | *P-value* |
| EF (HFmrEF vs. HFrEF) | -0.01 (-0.22 – 0.20) | 0.952 | -0.06 (-0.28 – 0.16) | 0.594 | -0.04 (-0.26 – 0.18) | 0.728 |
| EF (HFpER vs. HFrEF) | 0.11 (-0.05 – 0.28) | 0.163 | -0.03 (-0.20 – 0.14) | 0.737 | 0.02 (-0.14 – 0.18) | 0.798 |
| Observations | 312 | | 312 | | 312 | |
| R^2^ | 0.139 | | 0.152 | | 0.180 | |
| PASS – patient acceptable symptom state; KOOS – knee disability and osteoarthritis outcome score; CI – confidence interval; EF – ejection fraction; HFrEF – heart failure with reduced ejection fraction; HFmrEF – heart failure with mildly reduced ejection fraction; HFpEF – heart failure with preserved ejection fraction. | | | | | | |
